# Distinctive Genome Reduction Rates Revealed by Genomic Analyses of Two *Coxiella-*Like Endosymbionts in Ticks

**DOI:** 10.1093/gbe/evv108

**Published:** 2015-05-28

**Authors:** Yuval Gottlieb, Itai Lalzar, Lisa Klasson

**Affiliations:** ^1^Koret School of Veterinary Medicine, The Robert H. Smith Faculty of Agriculture, Food and Environment, The Hebrew University of Jerusalem, Rehovot, Israel; ^2^Molecular Evolution, Department of Cell and Molecular Biology, Uppsala University, Sweden

**Keywords:** symbiosis, genome reduction, *Coxiella*

## Abstract

Genome reduction is a hallmark of symbiotic genomes, and the rate and patterns of gene loss associated with this process have been investigated in several different symbiotic systems. However, in long-term host-associated coevolving symbiont clades, the genome size differences between strains are normally quite small and hence patterns of large-scale genome reduction can only be inferred from distant relatives. Here we present the complete genome of a *Coxiella*-like symbiont from *Rhipicephalus turanicus* ticks (CRt), and compare it with other genomes from the genus *Coxiella* in order to investigate the process of genome reduction in a genus consisting of intracellular host-associated bacteria with variable genome sizes. The 1.7-Mb CRt genome is larger than the genomes of most obligate mutualists but has a very low protein-coding content (48.5%) and an extremely high number of identifiable pseudogenes, indicating that it is currently undergoing genome reduction. Analysis of encoded functions suggests that CRt is an obligate tick mutualist, as indicated by the possible provisioning of the tick with biotin (B7), riboflavin (B2) and other cofactors, and by the loss of most genes involved in host cell interactions, such as secretion systems. Comparative analyses between CRt and the 2.5 times smaller genome of *Coxiella* from the lone star tick *Amblyomma americanum* (CLEAA) show that many of the same gene functions are lost and suggest that the large size difference might be due to a higher rate of genome evolution in CLEAA generated by the loss of the mismatch repair genes *mutSL*. Finally, sequence polymorphisms in the CRt population sampled from field collected ticks reveal up to one distinct strain variant per tick, and analyses of mutational patterns within the population suggest that selection might be acting on synonymous sites. The CRt genome is an extreme example of a symbiont genome caught in the act of genome reduction, and the comparison between CLEAA and CRt indicates that losses of particular genes early on in this process can potentially greatly influence the speed of this process.

## Introduction

Symbiotic microorganisms have been found in almost all arthropod lineages, where they can induce a multitude of different phenotypes, including, for example, defense against pathogens and changing of host sex ratios and behavior (Buchner 1965; Moran et al. 2008). However, in arthropods that live on unbalanced diets, such as plant sap or vertebrate blood, mutualistic microbes are especially common and thought to contribute the metabolites that are lacking from their hosts diet (Hansen and Moran 2014; Nikoh et al. 2014). As these nutritional mutualists are necessary for the reproduction and survival of their hosts, that is, obligate, the infection generally becomes fixed in the host population and a mode of vertical transmission is established.

Several genomic traits, such as low GC-content and high substitution rates, have been associated with genomes of endosymbionts, but genomic reduction is probably the most consistent and pronounced (Moran and Bennett 2014). The process of genome reduction is initiated as a consequence of loss of selection on multiple gene functions when a free-living bacterium becomes host-associated (Toft and Andersson 2010). As the environment within a host, particularly inside a host cell, is relatively stable and nutrient rich, the need for regulation, secondary metabolite biosynthesis and defense is largely lost, and over time so are the genes encoding such functions. For obligate mutualists, this process can continue even further, leading to tiny genomes where even genes considered essential are lost (Moran and Bennett 2014). Over time, obligate mutualistic interactions lead to coevolution between the partners, as can be seen when comparing phylogenetic trees of hosts and their symbionts (Vavre and Braig 2012).

Host-associated lineages can be found in basically all bacterial phyla, but there are a few orders that seem to be almost exclusively made up of them (Toft and Andersson 2010). One such order is the Legionellales within the gamma-Proteobacteria, which consists of the two families Legionellaceae and Coxiellaceae. Legionellaceae mainly contains a number of species in the genus *Legionella*, and although often found in the environment, it is only within a host that they replicate and divide (Fliermans 1996). The Coxiellaceae family contains a few genera, of which *Coxiella* and *Rickettsiella* are the best known, and all species in these genera have been considered obligate intracellular symbionts (Roux et al. 1997).

Within the genus *Coxiella*, almost all studies are based on a single species, *Coxiella burnetii*. It is the causative agent of Q-fever and endocarditis in humans (Seshadri and Samuel 2005), but its main reservoirs are other animals such as sheep, cattle, and goats. Although, *C. burnetii* has also been isolated from ticks (Maurin and Raoult 1999), direct transmission from ticks to humans has not been documented and probably plays a less important role than other means of disease transmission such as inhalation (Sprong et al. 2012). Like *Legionella* spp., *C. burnetii* is also readily found in the environment (Kersh et al. 2010), where it can persist for long periods as a metabolic quiescent small-cell variant (SCV). The SCV transitions to a metabolically active large-cell variant after the formation of the acidic parasitophorous vacuole (PV), the preferred niche of *C. burnetii* in the host cell (van Schaik et al. 2013).

In the last decade, *Coxiella*-like symbionts have been identified in various tick species (Noda et al. 1997; Klyachko et al. 2007; Zhong et al. 2007; Lalzar et al. 2012; Liu et al. 2013; Duron et al. 2014). As these symbionts are found in high prevalence in the host population and can be vertically transmitted from females to offspring (Clay et al. 2008; Machado-Ferreira et al. 2011; Lalzar et al. 2014), they are believed to be obligate tick symbionts. The observed cocladogenesis of host and symbiont (Almeida et al. 2012; Wilkinson et al. 2014) provides further evidence for this. So far, only one study has been conducted in order to elucidate the role of a *Coxiella*-like symbiont in the host (Zhong et al. 2007), but no specific functional contributions are yet known. However, as ticks are obligate blood feeders, a diet low in B-vitamins, provisioning of B-vitamins to the host would be a likely function for these obligate mutualists. The recently published full genome sequence of the *Coxiella*-like endosymbiont from a laboratory culture of the lone star tick *Amblyomma americanum* (termed CLEAA) supports this idea, as the CLEAA genome is only 657 kb, but has retained many genes involved in B vitamin and cofactor biosynthesis (Smith et al. 2015).

In this study, we present the complete genome sequence of a *Coxiella*-like symbiont from the tick *Rhipicephalus turanicus*, hereafter CRt for *Coxiella* of *Rh. turanicus*. Previous work has shown that the prevalence of CRt in *Rh. turanicus* in Israel is 100% (Lalzar et al. 2012) and that CRt is present at high densities within a host-derived membrane in Malpighian tubules of both males and females and in ovaries with a specific preference for the oocyte (Lalzar et al. 2014), indicating CRt is likely a vertically transmitted obligate mutualist in this tick. To overcome potential biases related to laboratory-reared populations, cell culture, or cultivation, we sequenced the genome of CRt cells that were purified directly from a natural tick population. We show that although the CRt genome is relatively large compared with the CLEAA genome and most other obligate mutualistic bacteria, its encoded functions are massively eroded which leaves a genome with a coding capacity of only 48.5%. By comparing the two tick symbiont genomes with several genomes of the pathogenic species *C. burnetii* and other Legionellales, we also pinpoint functions that might have been lost in the process of becoming an intracellular mutualist of ticks. Furthermore, we demonstrate that the CRt population sampled from noncultured ticks is heterogeneous; with up to one distinct strain variant per tick, and that the pattern of mutations might suggest selection on synonymous sites. Finally, we suggest that the large difference in genomes size between CRt and CLEAA might be a result of the loss of the mismatch repair genes *mutSL* in CLEAA.

## Materials and Methods

### Enrichment of *Coxiella* Cells and DNA Extraction

*Rhipicephalus turanicus* female ticks were collected at the outskirts of kibutz Hulda, Israel, placed in 100% ethanol, and kept at −20 °C until further use. Malpighian tubules and gonads were dissected from nine ticks as these organs were previously shown to harbor a larger amount of *Coxiella* DNA compared with the salivary glands, gut and accessory reproductive organs (Lalzar et al. 2014). The dissected tissues were pooled in a sterile 1.5-ml tube containing 100 µl sterile double distilled H_2_O and homogenized using a sterile pestle. The homogenate was transferred into a new sterile tube containing 10 ml of sterile double distilled H_2_O and was incubated in room temperature for 1 h, after which it was filtrated through a sterile gauze pad and a Minisart 5-µm filter (Sartorius AG, Gottingen, Germany). The filtrate was centrifuged for 15 min at 20,000 × g at 4 °C, and the supernatant was carefully discarded. The remaining pellet was used for subsequent genomic DNA (gDNA) extraction as described in Lalzar et al. (2012). To verify that the enrichment procedure worked as expected, *Coxiella* densities were determined by quantitative polymerase chain reaction (qPCR) analysis as previously described (Lalzar et al. 2012).

### Library Construction and Sequencing

Construction of gDNA libraries and sequencing were performed at the University of Illinois at Chicago, DNA Services Facility. Extracted gDNA was processed for sequencing using the Nextera DNA Sample Prep Kit (Illumina-Compatible; Epicentre Biotechnologies, Madison, WI) according to the manufacturer’s instructions. Briefly, 50 ng of gDNA was used as starting material for the kit, and after purification, DNA fragments of 400–800 bp were selected using the PippinPrep automated electrophoresis platform (Sage Scientific, Beverly, MA). Libraries were quantified using the KAPA library quantification system (KAPA Biosystems, Cape Town, South Africa) and subsequently sequenced on an Illumina HiSeq2000 instrument. A total of 30 Gb of 2 × 100 bp paired-end sequence data was obtained.

### Assembly

The Illumina reads were initially assembled into contigs with the CLCBio software (Cambridge, MA) to create a draft genome sequence. The initial CLCBio assembly was then improved by running multiple assemblers, including AbySS (Simpson et al. 2009)*,* Velvet (Zerbino and Birney 2008) and SGA (Simpson and Durbin 2012), and their results were combined using Consed (Gordon et al. 1998) followed by manual curation in order to close gaps and determine the reliability of the assembled genome. The few final gaps and long repeats found in the resulting draft genome were then covered by PCR. PCR products were cloned and sequenced as previously described (Lalzar et al. 2012). Additionally, a large duplication was discovered when mapping the Illumina reads against the assembled genome with Burrows-Wheeler Aligner (BWA) (Li and Durbin 2009), as it had double the coverage compared with the rest of the sequence. As this region was too large to PCR across, primers were designed from unique sequence into the repeat on both ends to confirm its position in the genome and from the end of the repeat to the beginning of the repeat to confirm that the repeat was present in tandem. The resulting PCR products were sequenced directly using the Sanger method. The complete genome of *Coxiella* sp. CRt is deposited in GenBank under accession number CP011126.

### Annotation

The annotation was performed by running a pipeline created in the DIYA framework, as described in Ellegaard et al. (2013), which includes gene calling by Prodigal (Hyatt et al. 2010), tRNA prediction by tRNAscan-SE (Lowe and Eddy 1997), rRNA prediction by RNAmmer (Lagesen et al. 2007), and determination of pseudogenes with GenePrimp (Pati et al. 2010). To assign functions, all putative protein-encoding genes were searched against the Uniprot database using BLASTp and pfam-scan was used to determine the presence of conserved protein domains found in PFAM (Bateman et al. 2004). The data were visualized and manually curated using Artemis (Rutherford et al. 2000). In addition, after manual curation, the protein-coding genes were used for metabolic analysis by KAAS (Moriya et al. 2007) and BLASTp was used to identify Clusters of Orthologous Groups (COG) categories. Nucleotide sequences of pseudogenes were analyzed in a similar way but using BLASTx. A gene was assigned to a COG if the *e* value of the best hit was less than 1e^−^^2^, and the top two hits were from the same COG.

### Phylogenetic Reconstruction

The annotated proteins from the CRt genome were clustered together with 11 other proteomes from the Legionellales order and seven outgroup species using OrthoMCL (Li et al. 2003) with an inflation value of 1.5. Accession numbers for the genomes included in the protein clustering are found in supplementary table S1, Supplementary Material online. The 285 single copy orthologous proteins found in all clustered genomes were aligned using mafft-linsi (Katoh et al. 2002) and sites with more than 50% gaps were removed after which the individual alignments were concatenated. A species tree (supplementary fig. S1*A*, Supplementary Material online) was inferred with RAxML version 8.1.16 (Stamatakis 2006) from the concatenated alignment using the PROTGAMMALG model with empirical amino acid frequencies, which was selected as the best model using the PROTGAMMAAUTO parameter, and constructed with one slow best maximum-likelihood tree and 1,000 rapid bootstrap replicates.

Proteins only found in CRt but absent in all other Legionellales genomes used in the OrthoMCL clustering were searched against the nonredundant database at National Center for Biotechnology Information using BLASTp. The 30 best hits with an *E* value less than 1e^−^^5^ and 30% or higher identity were used to reconstruct a phylogenetic tree using the same methods as described above for the species tree. All phylogenetic trees were visualized using FigTree (http://tree.bio.ed.ac.uk/software/figtree/, last accessed June 11, 2015).

### Repeats, Gene Order Comparison, and Gene Maps

Tandem repeats were detected using the Tandem repeat finder (Benson 1999) with the recommended settings, and a maximum period size of 500 bp. Larger, distributed repeats were detected using the MUMmer 3 package (Kurtz et al. 2004).

The CRt, CLEAA, and *C. burnetii* RSA493 genomes were searched against each other using tBLASTx and increasing –max_hsps_per_subject to 5,000, in order to visualize most of the homologous regions between them. The output from tBLASTx was then used to generate the genome comparison maps (fig. 2 and supplementary fig. S1, Supplementary Material online) with the genoPlotR package (Guy et al. 2010). Whole-genome alignments were done using Mauve (Darling et al. 2004) with one of the copies of the large duplication in CRt masked, and the resulting permutations file was used as input for MGR (Multiple Genome Rearrangements) (Bourque and Pevzner 2002), with the circular genome setting, to infer the ancestral gene order and phylogenetic tree.

### Variant Calling

The Illumina reads were cleaned from adapters and quality trimmed by Trimmomatic (Bolger et al. 2014), overlapping reads from short fragments were joined using seqprep and the resulting paired-end and merged reads were mapped against the genome using the BWA (Li and Durbin 2009) algorithm with default parameters. The Picard toolkit (http://broadinstitute.github.io/picard/, last accessed June 11, 2015) was used to convert the output from sam- to bam-format, sort the read according to coordinates and mark duplicate reads. The indel realigner from GATK was used to improve the alignment of reads and the GATK-unified genotyper was used to call variants, using a ploidy level of 4. Single nucleotide polymorphism (SNP) and indel calls were separated and GATK best practice filters were applied to eliminate false positives. The resulting variant calls were used in snpEff (Reumers et al. 2005) to get the functional annotation of variants.

## Results

### General Features of the CRt Genome

The CRt genome was sequenced to approximately 5,000× coverage with 100-bp paired-end Illumina reads, which after initial assembly followed by merging of assemblies and PCR gap closure resulted in a circular genome (see Materials and Methods). However, when mapping the reads to our initial circular genome we detected a large region (∼147,480 bp) with two times higher coverage than the rest of the genome. After performing PCR over the edges of this region (see Materials and Methods), we concluded that the two copies of this large repeat are likely present in tandem. A GC-skew plot over the genome gives further support for this assumption, as the two halves of the chromosome are more equal in size when this region is duplicated (data not shown). Given the large size of this repeat, we were unable to further differentiate between the two copies, but based on the mapped reads we have hypothesized that the two copies are identical to each other (but see also Genetic variation in CRt) and they are hence presented as such in the CRt genome deposited in the databases. The complete genome sequence of CRt thus consists of a single circular chromosome of 1,733,840 bp with 912 predicted protein-coding genes and as many as 675 putative pseudogenes, resulting in a very low coding content of only 48.5% (table 1). As the duplicated region contains 87 protein-coding genes (supplementary table S2, Supplementary Material online), the number of unique annotated protein functions in the CRt genome is as low as 825. The genome also contains one complete rRNA operon and 49 tRNAs, with potential for being charged with all 20 standard amino acids. Apart from the large duplicated region, the CRt genome contains very few repeated sequence, with only ten repeated regions larger than 100 bp making up a total of 8,611 bp. None of these repeats corresponds to known mobile elements, and only a single pseudogenic transposase is found.

**Table 1:** Genome Features of *Coxiella* of *Rhipicephalus turanicus* (CRt), *Coxiella* of *Amblyomma americanum* (CLEAA), *Coxiella burnetii* RSA493 (Cb), *Mycobacterium leprae* (ML), *Sodalis glossinidius* (SG), *Serratia symbiotica* of *Acyrthosiphon pisum* (SAp), *Serratia symbiotica* of *Cinara tujafilina* (SCt), and *Serratia symbiotica of Cinara cedri*

Feature	CRt	CLEAA	Cb	ML	SG	SAp	SCt	SCc
Genome size	1.7	0.66	1.99	3.27	4.17	2.79	2.49	1.76
GC (%)	38.2	34.6	42.6	57.59	54.7	48.4	52	29.22
Protein coding (%)	48.5	83.9	89.1	49.5	50.9	56.8	53.4	38.8
Genes	912	551	2,094	1,604	2,432	2,098	1,601	672
Pseudogenes	675	10	83	1,116	972	550	916	59

### Genome Dynamics of CRt

**Fig. 1.—:**
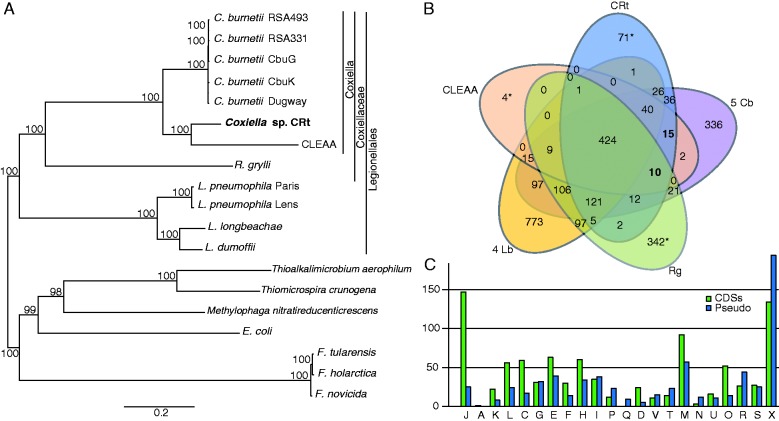
Phylogenetic relationship and protein clusters of 12 genomes from the order Legionellales. (*A*) The phylogenetic tree was inferred using maximum likelihood from a concatenated alignment of 285 single copy orthologous genes. The numbers on each node represent the support of 1,000 bootstrap replicates. (*B*) Venn diagram depicting numbers of protein clusters in five specified groups or genomes, Rg, *Rickettsiella grylli*; 5 Cb, All 5 *C. burnetii *genomes; 4 Lb, all four *Legionella *genomes. *includes both clusters and single proteins not found in any cluster. (*C*) A comparison between COG assignment for coding genes (green) and pseudogenes (blue) found in CRt. A: RNA processing and modification; J: translation, ribosomal structure and biogenesis; K: transcription; L: replication, recombination, and repair; C: energy production and conversion; M: cell wall/membrane biogenesis; D: cell cycle control, cell division; F: nucleotide transport and metabolism; H: coenzyme transport and metabolism; G: carbohydrate transport and metabolism; O: posttranslational modification, protein turnover, and chaperones; U: intracellular trafficking; T: signal transduction mechanisms; I: lipid transport and metabolism; V: defense mechanisms; P: inorganic ion transport and metabolism; Q: secondary metabolites biosynthesis, transport, and catabolism; N: cell motility; R: general function predicted only; S: function unknown; X: not assigned.

To investigate the dynamics of gene loss and gain in the CRt genome, clustering of orthologous proteins was performed using the proteomes of CRt and 11 other Legionellales and 7 outgroup species. A robust phylogeny created based on the 285 single copy orthologs found in all the species clearly shows the monophyletic relationship between CRt, CLEAA, and *C. burnetii*, to the exclusion of *Rickettsiella* (fig. 1*A*) and confirms that CRt is indeed a species in the genus *Coxiella*. The two tick symbionts, CRt and CLEAA, group together to form a monophyletic clade, although with CLEAA on a much longer branch than CRt indicating a faster rate of evolution in this symbiont.

An analysis of the distribution of protein clusters among families and genera demonstrated a very low number of protein-coding genes specific to the Coxiellaceae (fig. 1*B*), with only ten protein clusters uniquely present in all Coxiellaceae members. Within Coxiellaceae, no proteins are uniquely shared between the two tick symbionts CRt and CLEAA and as few as two protein clusters are exclusive to CRt and *R. grylli*, whereas 15 are shared between all three *Coxiella* species, and 36 and 2 clusters are uniquely shared between CRt and all five *C. burnetii* strains or CLEAA and all five *C. burnetii* strains, respectively (fig. 1*B*).

Of the 15 protein clusters unique to the genus *Coxiella*, as many as seven represent functions that are present in both *Coxiella* and *Legionella* (and sometimes also in *Rickettsiella*), but the enzymes have either different origin and/or are of different types. These include two genes involved in isoprenoid biosynthesis that were previously shown to be horizontally transferred (Gophna et al. 2006), the enzymes fructose-bisphosphate aldolase, 3-dehydroquinate and pantothenate kinase, as well as *bioC* and *csrA* that have paralogs both within *Coxiella* and *Legionella*. The remaining eight clusters contain two hypothetical proteins, an aminopeptidase, a kinase, a reductase, a pyrimidine nucleotide-disulfide oxidoreductase, cardiolipin synthetase, and uracil-DNA-glycosylase. However, not a single gene conserved within this genus is unique, as all of the genes are indeed present in other species outside of our comparison. This might not be very surprising, given that all of the *Coxiella* genomes are reduced, and particularly the tick endosymbionts CRt and CLEAA have very small proteomes.

Despite the close relationship between CRt and *C. burnetii*, 669 protein clusters were conserved in all five *C. burnetii* strains and various other members of the Legionellales, but were absent in CRt. Out of these, 353 were identified as pseudogenes in the CRt genome, whereas 314 were completely absent (table 2). The ratio of pseudogenes to absent genes in the CRt genome among these 669 clusters clearly reflects the phylogenetic level of conservation of the gene. For example, only 12 of the 106 genes present in all other Legionellales except CLEAA are completely absent in CRt, whereas 238 of the 336 genes uniquely conserved in *C. burnetii* are missing in CRt (table 2). A higher level of conservation within the Legionellales likely indicates loss in CRt, as compared with gain in the other lineages. Hence, there are still traces in CRt, in the form of pseudogenes, of genes that are highly conserved and likely have important and selected functions in other Legionellales species. In contrast, the smaller genome of CLEAA has already completely lost most of the functions that are currently present as pseudogenes in CRt, regardless of the level of conservation with the Legionellales. This clearly indicates that CRt and CLEAA have gone down a similar road of reduction, losing many of the same functions, although at seemingly different rates.

**Table 2:** Pseudogenes versus Lost Genes in CRt at Different Levels of Phylogenetic Conservation

Conservation Level	Total	Pseudo CRt	Absent CRt	Present CLEAA	Absent %
Legionellales	114	101	13	8	11.4
*Coxiella burnetii* and *Legionella*	112	91	21	15	18.8
Coxiellaceae	21	14	7	0	33.3
*Coxiella burnetii* and ≥1 *Legionella*	84	45	39	3	46.4
*Coxiella burnetii*	338	100	238	2	70.4

**Fig. 2.—:**
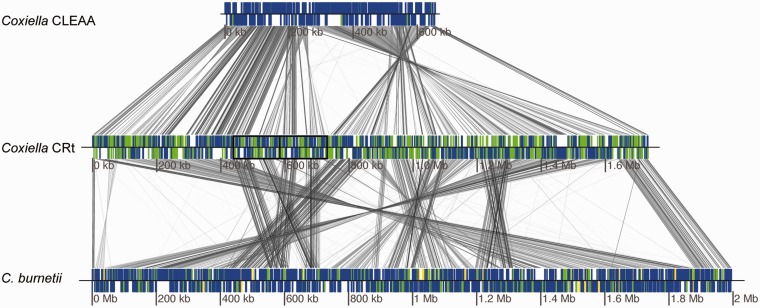
Genome comparison of CLEAA, CRt, and *C. burnetii.* Blue boxes depict protein-coding genes, green boxes represent pseudogenes, and yellow boxes indicate transposases. The regions framed in black indicate the position of the large duplicated region in CRt. Similarity between genome is shown by the gray lines, where a darker shade indicates higher similar.

To get an overall view of the level of synteny between the *Coxiella* genomes, and as gene order conservation is a good indicator of orthology, we aligned the whole genomes of five *C. burnetii* strains and the CRt and CLEAA genomes (fig. 2, showing only one of the *C. burnetii *genome). As can be seen, the global gene order is not highly conserved between any of the three genomes, and the number of syntenic blocks (as identified by Mauve) ranges from 30 between CLEAA and CRt to 57 between CRt and *C. burnetii.*

Even so, complete loss of DNA in CLEAA is generally observed in syntenic genomic regions where the CRt genome contains pseudogenes with functional orthologs in *C. burnetii *(supplementary fig. S1, Supplementary Material online), further indicating that CLEAA and CRt have lost the same functions at different rates.

Additionally, we reconstructed the putative ancestral gene order of the *Coxiella* genus and inferred a phylogeny (supplementary fig. S2, Supplementary Material online). Interestingly, the tree is in high agreement with the phylogenetic tree made from orthologous proteins, and again shows that CLEAA is located on a long branch with as many as 13 unique genomic rearrangements.

### Loss of Basic Cellular Functions in Tick Symbionts

Dramatic differences in the rate of pseudogenization of protein-coding genes among different functions, represented by COG categories, can be seen in the CRt genome (fig. 1*C*). As expected, the rate was lowest in the categories for basic cellular processes such as translation and transcription, whereas most genes related to functions such as cell motility and secondary metabolites biosynthesis, transport, and catabolism were pseudogenized.

Even so, a few CRt losses can be found among the set of highly conserved genes in Legionellales encoding basic cellular functions. Several genes involved in the modification of tRNA are pseudogenized in the CRt genome, including two genes required for the conversion of guanine to queuosine in the wobble position of GUN anticodons, *tgt *and *queA* (Noguchi et al. 1982). The queuosine modification is assumed to increase accuracy of translation, and change the optimal binding from codons ending in C to no preference or a slight preference for U-ending codons for GUN codons (Ran and Higgs 2010). Interestingly, all tRNAs with GUN anticodons are present in CRt; however, codons with T at third position outnumber the ones with C (approximately 0.25 C) in its genome, indicating that CRt might be losing translation efficacy and accuracy. Additionally, three other tRNA modifiers, *sun, trmJ,* and *dusA*, are pseudogenized in the CRt genome. In CLEAA, all these genes have been completely lost and one additional tRNA modifier *truB*, conserved in all other genomes, is also missing. Loss of genes involved in tRNA modifications is common among small genomes of obligate endosymbionts (de Crécy-Lagard et al. 2012).

Additionally, among the genes with basic cellular functions conserved in all Legionellales, CRt has lost three transcriptional regulators; one of the sigma factors, *rpoS *(stress response) and the two regulators *oxyR, *the oxidative stress regulator, and *nrdR*, a repressor of ribonucleotide reductases. Again, all of these genes are also absent from the CLEAA genome, together with a few additional transcriptional regulators.

Moreover, among the 119 genes putatively lost in CLEAA, as they are preserved in all other Legionellales, many are involved in replication, DNA repair, and chromosome segregation and condensation. For example, DnaA, the protein that initiates DNA replication, together with its regulator Hda are both missing (Mott and Berger 2007), as well as the genes encoding one of the subunits of DNA polymerase (*dnaXZ*), Topisomerase IV (*parCE*), and the Integration host factor (*ihfAB*). Furthermore, Fis, involved in organization and maintenance of nucleoid structure, as well as the three genes of the structural maintenance of chromosomes (SMC) complex, *smc* and *scpAB* together with the cell division protein FtsK are all absent from CLEAA (Gruber 2014). Additionally, two DNA repair systems, the mismatch repair system (*mutSL*) (Schofield and Hsieh 2003) and the nucleotide excision repair (*uvrABCD*) (Kisker et al. 2013) have been lost in CLEAA. Taken together these losses indicate that CLEAA might have lost its ability for independent initiation of DNA replication, as well as several important genes involved in chromosome segregation and DNA repair.

### Metabolic and Biosynthetic Capabilities of CRt

Similar to *C. burnetii, *CRt contains all genes for the TCA cycle and glycolysis except the first step involving the conversion of glucose to glucose-6-phosphate, suggesting that CRt can use carbohydrates as a carbon source for energy production (supplementary fig. S3, Supplementary Material online). However, the putative glucose transporter CBU_0265 of *C. burnetii *(Seshadri et al. 2003) is pseudogenized in CRt, so it is unclear how CRt would obtain glucose. CRt is likely able to produce ribose-5-phosphate through the pentose phosphate pathway, but like in *C. burnetii,* it lacks glucose-6-phosphate dehydrogenase and 6-phosphogluconate dehydrogenase, indicating that CRt cannot produce NADPH through this pathway. All complexes of the oxidative phosphorylation chain are present, including a complete ATP synthase and the cytochrome bo oxidase genes (*cyoABCDE*) (supplementary fig. S3, Supplementary Material online). However, the cytochrome bd oxidase genes that function under limiting oxygen levels (*cydAB*) are both pseudogenized in CRt and absent in CLEAA whereas present in *C. burnetii *and the *Legionella* species. Additionally, all genes responsible for the degradation fatty acids through the beta-oxidation pathway are pseudogenized in CRt and missing in CLEAA, which means that CRt and CLEAA might not utilize fatty acids for energy production (supplementary fig. S3, Supplementary Material online). Taken together, this suggests that the main mode of energy production in CRt and CLEAA is through aerobic respiration. This is in contrast to *C. burnetii, *where recent studies have shown a requirement for microaerophilic conditions for optimized growth in axenic culture (Omsland and Heinzen 2011).

Auxotrophy for amino acids is characteristic of the *Coxiella* genus, and both *C. burnetii *and *Legionella *species are thought to utilize amino acids as their main carbon source (Omsland and Heinzen 2011). Like *C. burnetii* (Seshadri et al. 2003), CRt is predicted to be auxotrophic for 11 amino acids and hence compensation by exogenous supply of amino acid or oligopeptides is necessary. Other members of the *Coxiella* genus use amino acid and oligopeptide transporters and permeases that scavenge amino acids from the host (Fuchs et al. 2012) but compared with *C. burnetii*, CRt shows a reduction in these compensating routes which is even further exaggerated in the CLEAA genome. For example, the arginine and methionine ABC transporters present in *C. burnetii *are absent or pseudogenized in both tick symbiont genomes, and several amino acid permeases are pseudogenized in CRt and absent in CLEAA. Furthermore, the CRt genome contains ten pseudogenes of major facilitator superfamily (MFS) transporters that have documented roles in amino acid uptake (Chen et al. 2008; Beare et al. 2009) and an additional five losses of MFS transporters were inferred in CLEAA.

CRt has lost the ability for de novo synthesis of purines, but has retained the purine salvage pathway and the de novo pathway for synthesis of pyrimidines. Interestingly, the smaller genome of CLEAA has retained the genes for de novo synthesis of both purines and pyrimidines (supplementary fig. S4, Supplementary Material online).

### Putative Symbiont Functions

Even though rarely demonstrated, obligate blood feeding arthropods such as tsetse flies, bedbugs, louse, and presumably ticks are considered to be dependent on symbionts for the provision of B vitamins and cofactors, as those are rare in vertebrate blood (Zhong et al. 2007; Hosokawa et al. 2010; Kirkness et al. 2010; Snyder et al. 2010; Smith et al. 2015).

**Fig. 3.—:**
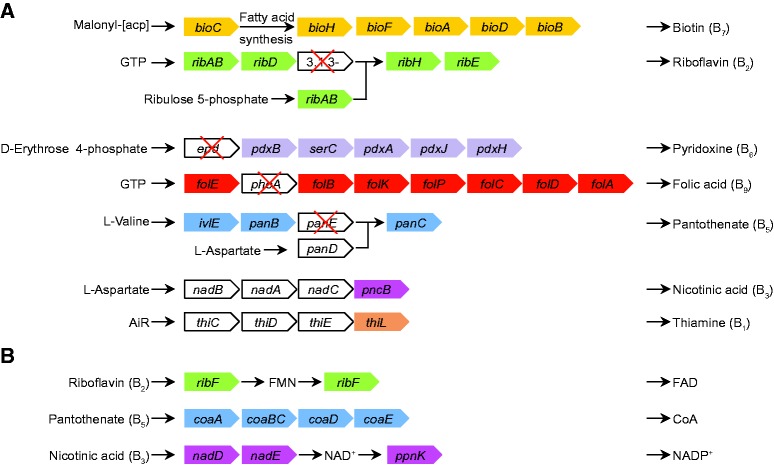
Biosynthetic pathways for B vitamins (*A*) and cofactors (*B*) in CRt. Gene names are indicated in colored rectangles. White rectangles indicate pseudogenes. White rectangles with red X indicate a missing gene.

Genes involved in the production of seven B vitamins are found in the CRt genome (fig. 3), but the completeness of the various pathways varies greatly. The pathways for the synthesis of biotin (B7) and riboflavin (B2), and the cofactors CoA, FMN and FAD are completely retained, as also seen in the genomes of a number of other symbionts of blood-feeding arthropods (Akman et al. 2002; Dunning Hotopp et al. 2006; Nikoh et al. 2014). The pathways for biosynthesis of pyridoxine (B6), folic acid (B9), and pantothenic acid (B5) are each missing a single gene (*epd*, *phoA,* and *panE,* respectively; fig. 3). As seen for the pyridoxine biosynthesis in other species such as *Bacillus stearothermophilus *and *Escherichia coli*(Yang et al. 1998), it is possible that the function encoded by *epd* is instead performed by GapA or another noncanonical enzyme hence yielding a complete pathway in CRt. Similarly for the folate biosynthesis, the function encoded by *phoA* might be performed by FolE as suggested for *Wigglesworthia *(Akman et al. 2002) or PhoA may not be required at all (Bermingham and Derrick 2002). For the biosynthetic pathway of pantothenic acid (B5), it was recently shown in *Francisella tularensis* that the gene *panG* could substitute the function encoded by *panE* (Miller et al. 2013). As an ortholog of *panG *is present in CRt, it might be able to synthesize vitamin B5 by the use of this gene. The biosynthetic pathways for these five B vitamins are identical between the two tick symbionts CRt and CLEAA. In contrast, the two pathways for the synthesis of thiamine (B1) and nicotinate (B3) are both highly degraded in CRt (fig. 3), whereas CLEAA has retained functional copies of all the pseudogenized genes in CRt. However, whereas the CRt genome contains a putative nicotinamide riboside transporter that might provide CRt with the precursor for B3 and NAD(P) from the host, this transporter has been lost in the CLEAA genome and thus retention of the full pathway might be necessary. Additionally, although CLEAA has retained all genes for thiamine biosynthesis that are degraded in CRt, it has lost the gene *thiL* performing the final step in converting thiamine phosphate to thiamine diphosphate, the form used as cofactor in many enzymes (Begley et al. 1999).

Iron is a critical component for cellular processes in both the host cell and bacteria (Collins 2003), and as a consequence they may compete for its acquisition. On the other hand iron excess, a probable scenario for blood feeding arthropods, can cause severe cell damage and may be lethal to both bacteria and the host (Schaible and Kaufmann 2004). Hence, symbionts of blood feeding arthropods can benefit from a rich iron environment and iron acquisition by these symbionts might in turn also be beneficial for the host. No genes involved in known iron scavenging systems were found in the CRt genome, but iron might be directly obtained by CRt through the FeoABC transporter in the form of ferrous iron (Fe^2+^) under both anaerobic and aerobic conditions (Kim et al. 2012). In addition, the *fur *gene, a global regulator of iron uptake, is also found in the genome. However, most of the iron in blood is bound in heme. It is likely that heme is imported by CRt, as all genes involved in heme biosynthesis except one are pesudogenized in CRt, but none of the previously described heme uptake systems (Contreras et al. 2014) could be detected in the CRt genome. Nevertheless, it is possible that one of the more generally annotated transporters is involved in heme uptake. For example in *E. coli*, the dipeptide ABC transporter is able to transport heme from the periplasm to the cell cytoplasm and the nickel-binding protein NikA can bind heme in the periplasm and function in heme transport (Braun and Hantke 2011).

The localization of CRt within the Malphigian tubules, the organ responsible for excretion of nitrogenous waste, suggests that recycling of nitrogenous waste could be a possible function for the symbiont. Recycling of this nitrogenous waste by symbionts could be beneficial for both the host and the symbionts, as the symbiont gets access to nitrogen and the host might be provided with useful nitrogenous compounds such as essential amino acids (EAAs) that are not present in their diet (Macdonald et al. 2012). An example is seen in the metabolic reconstruction of *Blattabacterium*, the cockroach endosymbiont, which indicated that nitrogen from urea and ammonia could be recycled into glutamate, using urease and glutamate dehydrogenase (GDH) (Lopez-Sanchez et al. 2009; Sabree et al. 2009). The major routes for nitrogen assimilation by bacteria are by conversion of ammonia into glutamate and glutamine by GDH and glutamine synthetase (GS) (Leigh and Dodsworth 2007). Both CRt and CLEAA have retained GDH but the GS gene is pseudogenized in CRt and absent in CLEAA. GDH is further dependent on the presence of NAD that can be synthesized by CLEAA but not by CRt unless provided with the precursor nicotinic acid (vitamin B3). For comparison, *C. burnetii *has retained both the GDH and GS genes.

### Host Interactions

The Dot/Icm secretion system is essential for the intracellular survival and replication of both *Legionella* and *Coxiella* as it prevents the fusion of the bacterial containing vacuole with the lysosome by the translocation of effector proteins to the host cell (Segal et al. 2005). All genes encoding the Dot/Icm secretion system as well as several of the putative or known *C. burnetii *effector proteins secreted by this system have been pseudogenized in CRt and are missing completely in CLEAA, indicating that both tick symbionts have lost the main system used for host communication in other Legionellales species. Additionally, all genes associated with the type IV pili are pseudogenes in CRt and absent in CLEAA. Although a pilus has not been observed in *C. burnetii*, this system has been speculated to participate in secretion of proteins translocated to the periplasmic space through the Sec system (Stead et al. 2013) as seen in *Francisella novocida *(Hager et al. 2006). Together, these losses suggest that neither CRt nor CLEAA are residing in phagosome-derived vacuoles like *C. burnetii* but instead are enclosed in another perhaps less hostile host-derived membrane compartment (Lalzar et al. 2014), as seen in other obligate mutualistic bacteria (Moran and Bennett 2014). As a further indication that CRt is not adapted to the acidic environment of the PV like *C. burnetii*, the Na^+^/H^+^ antiporter encoded by the six genes *shaABCDEFG *in the *C. burnetii* genomes and believed to play a role in pH homeostasis and survival in the PV (Seshadri et al. 2003) is absent from the CRt and CLEAA genomes. Instead, in the same genomic location as the *sha *genes in *C. burnetii*, CRt and CLEAA encode the Na^+^/H^+^ antiporter NhaA that functions mainly at high salinity and alkaline pH (Padan et al. 1989). As the *sha *genes are absent from all the other Legionellales genomes and NhaA present in all except *C. burnetii*, they were most likely acquired through horizontal gene transfer (HGT) in the *C. burnetii* lineage rather than lost in CRt and CLEAA.

Lipopolysaccharides (LPS) is one of the few established virulence factors of *C. burnetii* (Hackstadt 1990; van Schaik et al. 2013). This is evident when virulent isolates of *C. burnetii *(Phase I) are cultured for long periods by passage in cell culture, as they often experience changes in the structure of their LPS that leads to loss of infectivity (Phase II). *Coxiella burnetii* phase II isolates produce a shorter O-antigen chain and lack two of the unique sugars of phase I *C. burnetii *LPS (Denison et al. 2007). Although the genetic changes associated with phase variation in *C. burnetii* have not been fully determined, a large chromosomal deletion encompassing genes putatively involved in O-antigen biosynthesis has occurred in some phase II variants (Vodkin and Williams 1986; Hoover et al. 2002; Denison et al. 2007).

**Fig. 4.—:**
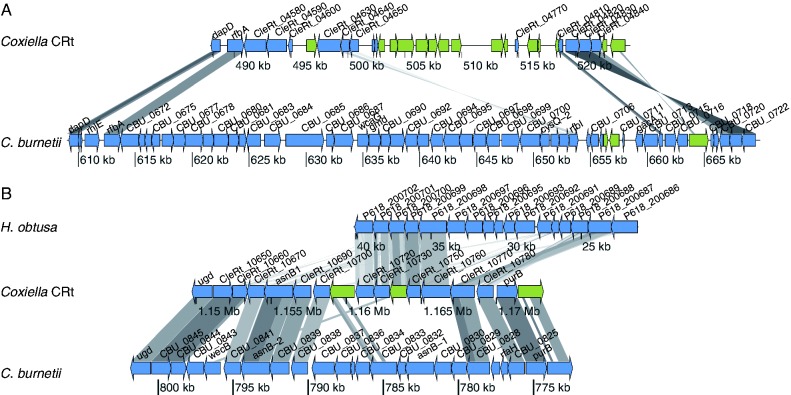
Comparison between CRt and *C. burnetii *of genomic regions putatively involved in O-antigen biosynthesis in *C. burnetii*. Blue arrows depict protein-coding genes and green arrows represent pseudogenes. Similarity between genes is shown by the grey lines, where a darker shade indicates higher similar. (*A*) Genomic region associated with deletion in *C. burnetii *Phase II isolates (Hoover et al. 2002). (*B*) Genomic region putatively involved in expression of sugars found in the O-antigen backbone (Seshadri et al. 2003). The genes unique to CRt are similar to *Holospora obtusa*, both in sequence and gene order.

Two loci putatively associated with O-antigen and LPS production have been identified in *C. burnetii *(fig. 4) (Seshadri et al. 2003). In the first locus, which is deleted in several phase II variants of *C. burnetii*, there is no similarity between the genes in CRt and *C. burnetii* (fig. 4*A*) even though several of the genes in CRt are also putatively associated with O-antigen biosynthesis and the flanking genes are homologous and conserved in order between the two genomes. As there are no orthologs in other Legionellales for the genes found at this locus in either of the two species (*C. burnetii *or CRt), it is not possible to determine whether either of them is ancestral, whether they were both independently gained by HGT, or whether the difference between them results from deletions in either or both of the two lineages. Additional database searches and phylogenetic reconstruction did not indicate any consistent and close relationships between the genes present at this locus in CRt and any particular taxon. Hence, it is not possible to draw any conclusion about the evolutionary scenario that generated these differences. Interestingly though, this locus is present within the large tandem duplication in CRt.

**Fig. 5.—:**
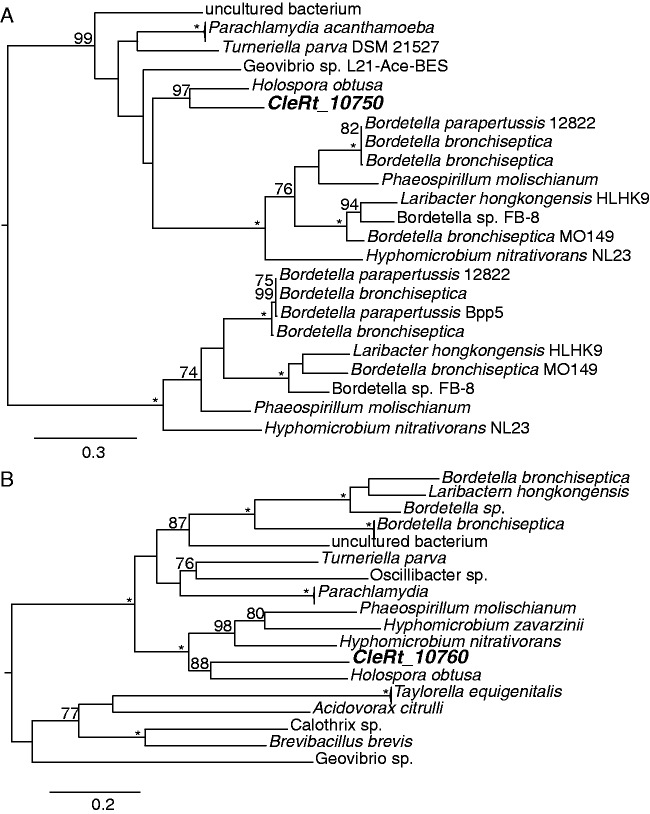
Phylogenetic analysis of genes putatively associated with an O-antigen locus in CRt. The phylogenetic trees were inferred based on protein alignments including CRt (in bold) and various other bacteria and using maximum likelihood. Numbers on the branches represent the support from 1,000 bootstrap replicates.

The second locus in *C. burnetii* has not been associated with phase variation but contains several genes normally associated with O-antigen biosynthesis (fig. 4*B*). Although several of the genes as well as the gene order is conserved between *C. burnetii* and CRt at this locus, nine of the genes contained in this locus in *C. burnetii* are missing or pseudogenized in CRt and instead the CRt genome contains four nonorthologous genes and one pseudogene. Phylogenetic analysis of the four genes shows that all of them consistently group together with various members of the alpha- and beta-proteobacteria (fig. 5). Although it is not possible to pinpoint the exact evolutionary origin of these genes, the phylogenetic reconstructions together with a conserved gene order of the locus in the genome of the alpha-proteobacterium *Holospora obtusa*, a macronuclear symbiont of the ciliate *Paramecium caudatum* (figs. 4*B* and 5), suggest that this region might have been horizontally transferred between these distantly related bacteria, although not very recently.

As both of these loci contain a substantial amount of pseudogenes in CRt, it is not very likely that CRt can produce a full-length O-antigen. However, even a short O-antigen chain could potentially be of relevance for interactions with host cells, such as inducing innate immune pathways, or increasing adherence and cell entry. None of the putative host interaction systems described here is present in the CLEAA genome, including the two LPS loci.

### Genetic Variation in CRt

Although the material used for genome sequencing was collected from relatively few individuals during limited time and space, the nonduplicated regions of the CRt genome contains at least 1,680 polymorphic sites, of which 1,481 are SNPs and 199 are indels.

**Fig. 6.—:**
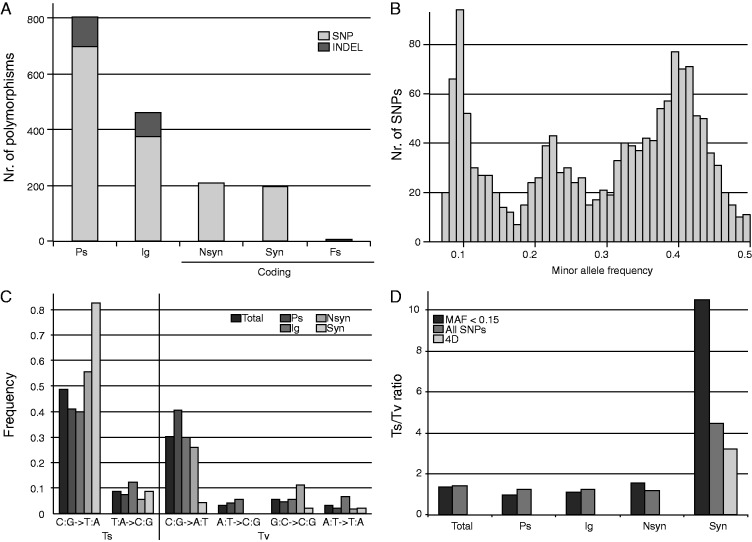
Polymorphisms in the genomic data of CRt. (*A*) Number and type of polymorphism found in coding and noncoding parts of the genome. Ps, pseudogenes; Ig, intergenic region; Nsyn, nonsynonymous; Syn, synonymous; Fs, frameshift. (*B*) Histogram of minor allele variants. (*C*) Mutational spectrum in different genomic regions inferred from variants with a frequency less than 0.15. (*D*) Ts:Tv ratio for low frequency allele variants and all allele variants, and variants at 4D sites.

As expected under selection, the majority of the polymorphisms is found in pseudogenes and intergenic regions (fig. 6*A*). However, a total of 407 SNPs can be found in genic sequences, with a relatively equal amount generating synonymous and nonsynonymous changes (fig. 6*A*). In order to investigate the number of stable variants present in the sample, the allele frequency for each SNP was calculated. The frequency of minor alleles, that is, the less common variants, varies among the SNPs, but three peaks are clearly observed in a histogram of the minor allele frequencies (MAF) (fig. 6*B*). Given that the sample represents a pool of bacterial DNA from nine ticks and the number of bacterial cells in each tick might not have been equal, it is not possible to determine the exact number of variants based on the MAF frequencies, but a minimum of three variants must be present to explain the data. Although the SNPs with an MAF around 0.1 could indicate the presence of even more stable variants, this peak might instead represent transient mutational variants.

An additional 148 SNPs and 19 indels are found in the large tandem duplication region when SNP calling is performed with one of the copies masked. These sites were not included in any further analyses, as it is not possible to definitely determine whether these polymorphic sites stem from variation between different CRt genomes or between the two copies of the repeat within the same genome. Even so, it should be noted that the frequency of variable sites (∼1 SNPs/1,000 bp) as well as the MAF distribution (supplementary fig. S5, Supplementary Material online) in the duplicated region is similar to the nonduplicated part of the genome, indicating that this region is evolving similarly to the rest of the genome.

As the sequence divergence between CRt and its closest relative CLEAA has reached saturation at synonymous sites, there is no reliable way of inferring the ancestral allele in the CRt population, and hence polarizing the SNPs to determine the direction of mutations. However, using only SNPs with an MAF lower than 0.15 in the sequenced population, and under the assumption that the direction of mutation is from the reference allele to the minor allele, the mutational spectrum of CRt was investigated. As seen in almost all organisms where neutral mutations have been investigated, the majority of mutations in CRt is from GC to AT, regardless of the location of the SNP (fig. 6*C*). However, compared with the other locations (Ps, Ig, and Nsyn) where C:G→A:T transversions (Tv) are almost as frequent as C:G→T:A transitions (Ts), synonymous mutations are heavily biased toward the transition mutation C:G→T:A.

To address these differences in mutational pattern, and as we have no rationale to infer the direction of the mutation when including all SNPs, we compared the Ts/Tv ratios among the different categories in our data set. The Ts/Tv ratio for pseudogenes, intergenic regions, and nonsynonymous sites is relatively similar to each other and close to 1, irrespective of whether using all SNPs (selection + neutral) or putatively transient SNPs with MAF < 0.15 (neutral), whereas the ratio at synonymous sites (fig. 6*D*) is much higher when using all SNPs and even further exacerbated for putatively transient mutations (4.47 all, 10.5 MAF < 0.15). Given that a majority of all possible synonymous mutations are transitions, the Ts/Tv ratio was also calculated for all mutations at 4-fold degenerate (4D) codon sites, where all possible mutations are synonymous. Although the Ts/Tv ratio at 4D codon sites is lower than when including all synonymous sites, the difference in mutational pattern at synonymous sites compared with other putatively neutral sites (intergenic and pseudogenic) is not completely explained by restrictions in the genetic code (fig. 6*C*), and might indicate that there is selection on synonymous sites in CRt.

Apart from the polymorphisms that were detected using the GATK pipeline (above), variability was seen in a majority of the 296 short tandem repeats (4–43 bp, 2-18 copies) in the CRt genome, based on manual inspection of read alignments. These types of repeats have previously been seen to vary between isolates of *C. burnetii*, and are part of the Variable Number Tandem Repeat (VNTR) marker system that has been developed for strain discrimination (Sabat et al. 2003). To investigate the polymorphisms in CRt, primers were designed over ten tandem repeat loci and PCR was then performed on eight adult ticks (four males and four females) collected from the same site and on the same dates as the ticks used for the gDNA extraction (see Materials and Methods). By comparing band migration sizes we were able to show that variation exists between individual ticks, but that different variants are not found within an individual tick (supplementary fig. S6, Supplementary Material online). Additionally, none of the eight ticks used in this VNTR analysis had exactly the same band migration pattern for all loci, indicating that every individual tick had its own CRt variant. As an extension of this observation, it is possible that the SNPs in the genomic data set could thus be a representation of up to nine CRt variants, as this is the number of ticks pooled together for the gDNA extraction.

### Suggestion for Species Name of CRt

The phylogenetic analyses based on orthologs of 12 genomes from the order Legionellales place CRt in a clade together with CLEAA within the genus *Coxiella*, but outside of the species *C. burnetii.* The two *Coxiella* branches represent different bacterial life histories, where *C. burnetii* is mostly found outside of tick hosts, either in the environment or in mammalian cells, whereas CRt and CLEAA are restricted to tick hosts and are most likely obligate mutualists. Based on our genomic analyses, and the difference between the two tick associated *Coxiella* we suggest naming CRt *Candidatus* Coxiella mudrowiae (mu.dro' wi.ae, N.L. gen. fem. n. mudrowiae) after Elizabeth Mudrow who first described symbiotic microorganisms in *Rhipicephalus* ticks in 1932 (Mudrow 1932).

## Discussion

At least three examples of bacteria with a coding content of approximately 50% or less exist in the literature, *Mycobacterium leprae* (Cole et al. 2001) *Sodalis glossinidius* (Toh et al. 2006), and *Serratia symbiotica *(Lamelas et al. 2011) (table 1). Although all three species are intracellular host-associated bacteria, they vary in both type of interaction with their host and genome size. Whereas *M. leprae* is a pathogen with very limited host range causing leprosy in humans, *So. glossinidius* and *Se. symbiotica* are both maternally transmitted endosymbionts of insects, tsetse flies and aphids, respectively. As *M. leprae *and *So. glossinidius* have relatively large genomes, their low coding densities have been speculated to be a result of recently adopting a host-restricted lifestyle (Toh et al. 2006). The genome of *Se. symbiotica* SCc, an obligate endosymbiont of *Cinara cedri*, has a genome size similar to CRt and a coding content of only 38.8% (Lamelas et al. 2011). However, in contrast to the large number of pseudogenes identified in CRt, SCc contains only 59 recognizable pseudogenes. Interestingly, another two *Se. symbiotica *genomes have been sequenced. The genome of SAp, a facultative endosymbiont of the aphid *Acyrthosiphon pisum*, is 2.79 Mb, and has a coding content of 60.9% and 550 pseudogenes (Burke and Moran 2011), whereas the genome of SCt, an obligate endosymbiont of the aphid *Cinara tujafilina*, is 2.5 Mb and has a coding content of 53.4% and 916 identified pseudogenes (Manzano-Marin and Latorre 2014). The differences in genome size and the level of gene degradation between the three *Se. symbiotica *genomes can largely be attributed to the closeness of the association with the host (obligate vs. facultative) and the level of genome reduction in the other obligate endosymbiont of the same aphid species, *Buchnera* (Manzano-Marin and Latorre 2014). Although a direct inference of symbiotic lifestyle cannot be made by comparing the characteristics of these genomes to the CRt genome, it is interesting to note that CRt has a genome size similar to the obligate symbiont SCc, a coding content that is intermediate to the two obligate symbionts and a higher ratio of identifiable pseudogenes to protein-coding genes than any of them.

It has previously been noted that *C. burnetii *have some features indicating ongoing genome reduction, including relatively high numbers of recent pseudogenes (Seshadri et al. 2003). Additionally, and in contrast to CRt, *C. burnetii *also has another common genomic mark of the early phase of host-association, expansion of repetitive and mobile elements (Seshadri et al. 2003; Beare et al. 2009) which is also believed to have contributed to the relatively high number of genomic rearrangements observed in the genomes of this species. However, CRt is clearly further along in the process of genome reduction than *C. burnetii*, as both the number and the level of degradation of pseudogenes are much higher and there appears to be no active mobile elements present in the genome. Furthermore, although gene content analysis supports the phylogenetic placement of CRt within the genus *Coxiella*, it is clear that divergence of CRt from *C. burnetii *is strongly marked by a much lower retention and/or gain of genes (fig. 1*B* and table 2). Our analysis indicates that a relatively large amount of genes might have been gained in the lineage leading to *C. burnetii*, as many genes were uniquely found in this species. As a further indication of this, we noted that several proteins with the same function had different evolutionary origin in *Coxiella *and *Legionella*. However, genome sequences from other species within the *Coxiella* genus will be needed in order to elucidate this further. The higher level of host-restriction and hence smaller population size, together with fewer interactions with other microorganism, might explain these genomic differences between *C. burnetii *and CRt.

The striking difference in genome size and thus in several metabolic and basic cellular functions between CRt and CLEAA might perhaps instead be explained by other factors. One possibility is that the transition to obligate tick symbiont has occurred multiple independent times in the *Coxiella *lineage. Under this scenario, CLEAA could have transitioned earlier and thus spent a longer time as an obligate tick symbiont than CRt, which might have resulted in a more reduced genome. However, as cospeciation between *Coxiella*-like endosymbionts and their tick hosts has been observed (Almeida et al. 2012; Wilkinson et al. 2014), and even though we cannot exclude the possibility that multiple origins of *Coxiella*-like symbionts might be undetected due to, for example, low sampling, these results indicate that this transition occurred only once and hence that CRt and CLEAA should have been tick-associated symbionts for the same amount of time. If so, the higher level of genome reduction in CLEAA might indicate a higher rate of evolution rather than a longer host-association as the cause of this difference. Although, substitution and deletion rates might not be determined by the same mechanisms, the longer branch of CLEAA in our phylogenetic analyses (fig. 1*A*) further suggests an increased rate of evolution in this lineage. Several factors might indeed influence the rate of evolution, including mutation rate, generation time, and population size (Bromham 2009; Weller and Wu 2015) as well as other host related factors such as presence of cosymbionts, life cycle, habitat or diet. Starting with the hosts, there are many similarities in the biology of the two tick species *A. americanum* and *Rh. turanicus,* including body size, a life cycle of three hosts and a wide range of possible hosts including wild and domesticated mammals, birds and humans (Ioffe-Uspensky et al. 1997; Childs and Paddock 2003). However, the two tick host species do differ in habitat; Whereas the North American lone star tick, *A. americanum*, is found in woodland habitats (Childs and Paddock 2003), the globally distributed *Rh. turanicus* is found in open land under moderate environmental conditions (Pegram et al. 1987; Mumcuoglu et al. 1993). Additionally, there are indications that the two tick species differ in generation time, as *Rh. turanicus* has one of the shortest generation times among Ixodidae ticks, and can potentially complete its life cycle in approximately 6 weeks (Ioffe-Uspensky et al. 1997), whereas it can take between approximately 21 weeks and up to 2 years or more for *A. americanum* under field conditions (Troughton and Levin 2007). If generation time of the host would be correlated to the generation time of the symbiont, this observation is completely counterintuitive as a shorter generation time (seen in *Rh. turanicus*) generally leads to a higher evolutionary rate, as more rounds of replication will generate more mutations over the same amount of time. Additionally, for symbionts, a faster generation time of the host will lead to a higher number of population bottlenecks due to maternal transmission of the symbionts to the next generation, which might also lead to a higher rate of evolution due to drift (Woolfit and Bromham 2003). However, it should be noted that CRt was sequenced from field collected ticks, whereas CLEAA was sequenced from laboratory maintained ticks. The different habitats and field versus lab conditions may result in different selectional pressure on the tick hosts, as well as affect generation time, but it is unclear how this would affect the evolutionary rate of the symbiont. A more likely explanation for the differences between CLEAA and CRt might instead come from the genomes themselves. Loss of DNA repair genes has been implicated in generating higher substitution rates in many symbionts genomes (Moran 1996; Moran et al. 2008), but interestingly, loss of the mismatch repair system encoded by *mutSL *has also been seen to result in a 50-fold increase in the rate of deletions (Nilsson et al. 2005). Additionally, as loss of *mutSL *is generally associated with a higher rate of intrachromosomal recombination (Petit et al. 1991), their loss could also have contributed to the higher number of unique genomic rearrangements that was inferred in the CLEAA genome. Although perhaps not the whole explanation, one possible scenario is thus that the loss of *mutSL *from the CLEAA genome might have resulted in a faster rate of both nucleotide substitutions, rearrangements and deletions, and hence over time a smaller genome with less noncoding DNA. Further genomes of *Coxiella*-like endosymbionts of ticks might help in verifying or rejecting this hypothesis.

Although the overall differences in genome size might be due to nonselective forces, some of the differences in metabolic functionality or host interaction might be a consequence of selection related to the specific host. There are notable differences between the two symbionts in their localization within the tick. CRt is mostly found in Malpighian tubules of both male and female *Rh. turanicus* ticks, in the female gonads and in very low densities in salivary glands (Lalzar et al. 2014). Similarly CLEAA is also found in Malpighian tubules and in the female gonads, but is also highly prevalent in *A. americanum* salivary glands (Klyachko et al. 2007). It is not known why CRt does not occupy the salivary glands in high densities, but the ability to invade different host tissues may be a result of specific *Coxiella*-host cell communication.

Interestingly, the differences in content of conserved genes between CRt and CLEAA to a very high extent mirror the differences between the two *Baumannia* strains BGSS and GWSS that are obligate mutualists of sharpshooters, although the genome size difference is much smaller between the latter two (approximately 75 kb and 89 protein-coding genes) (Bennett et al. 2014). The smaller genome of BGSS has, for example, lost *fis, ihfAB, dnaA *and the SMC complex, which are also missing in CLEAA. Additionally, similarity in losses between CLEAA and BGSS can also be found among the LPS core and putative O-antigen synthesis, as well as the Tol-Pal system used in cell envelope maintenance. Although convergence in the loss of functions during genomic reduction is known to occur between unrelated lineages of symbionts (Merhej et al. 2009), it is indeed striking to see such highly similar patterns. This observation makes it even more likely that many of the gene losses in CLEAA are not due to a specificity of host–symbiont interaction, but a general loss of selection on many functions when becoming an obligate mutualist.

The genome of CRt reveals possible functions relevant for the mutualism with its host, including the production of B vitamins and utilization of metabolites abundant in the host diet and secretions. As in other mutualists of blood feeding arthropods lacking B vitamins in their diet, it seems that CRt can produce at least five B vitamins B2, B5, B6, B7, and B9, which is less than CLEEA, that also encode genes for the biosynthesis of vitamin B1 and B3. However, experimental confirmation of these findings is still needed, as several of the pathways are missing one gene of the canonical pathway. Additionally, whether any of these is important for the symbiosis between CRt and its host is yet to be discovered.

The localization of CRt in specific organs in the tick body suggests that it is not exposed to the vertebrate blood and therefore does not need to cope with high heme concentrations as some other bacteria do (Graca-Souza et al. 2006; Roden et al. 2012). Additionally, a specific heme binding protein responsible for transporting the ingested heme directly to the hemosome for detoxification has been identified in the cattle tick *Rhipicephalus microplus* (Lara et al. 2005). Although not the same tick species, this further indicates that CRt might not be exposed to very high levels of heme that would require special adaptations.

Although the localization of CRt within the Malphigian tubules suggests that recycling of nitrogenous waste could be a possible function for the symbiont, ticks do not generally produce large amounts of the common nitrogenous waste products like urea and uric acid but instead produce crystals made of guanine and other purines, such as hypoxanthine and xanthine (Sonenshine and Roe 2014). Interestingly, their excretion products also commonly contain a large proportion of heme and/or hemoglobin from the ingested blood meal. As CRt is unable to produce purines and heme, localization of CRt within the Malphigian tubules may thus be a way for the symbiont to obtain these products rather than being related to the symbiotic function of CRt in its host.

It should be taken into account that in other mutualistic symbioses such as those in aphids and cockroaches, many essential pathways are shared between the symbiont and the host (Ramsey et al. 2010; Macdonald et al. 2012). As the genome of *Rh. turanicus* is not sequenced, it is too early to conclude the full contribution of CRt to its tick host.

Finally, polymorphisms have been found in several genome projects of endosymbiotic bacteria collected from field populations (van Ham et al. 2003; Van Leuven and McCutcheon 2012; Williams and Wernegreen 2012). Various observations have been made with regards to mutational patterns and number of variants, but to our knowledge the pattern of mutations between putatively nonselected sites (i.e., synonymous, intergenic and pseudogenic) have never been seen to differ in these studies. Although the cause for this difference is unclear, it might indicate that selection is acting on synonymous sites in CRt. Selection on synonymous sites is often attributed to selection on optimal codon usage that can increase both the rate and fidelity of translation (Sharp and Li 1987; Wald et al. 2012), but some studies also suggest that selectional constraints on chromosome structure might be a cause (Babbitt et al. 2014). DNA methylation can also affect both mutation rate and pattern in bacteria (Kahramanoglou et al. 2012; Lee et al. 2012) as particularly methylated cytosine is easily deaminated to thymine to yield a C→T transition. However, no DNA methylases have been found in the genome of CRt, and thus methylation status is likely not the cause of the pattern seen here. Future controlled population studies might shed light on the underlying cause of the contrasting mutational patterns between different sites in CRt, and whether this is a common trait among *Coxiella*-like symbionts.

In summary, we found that the genome of CRt is currently undergoing genome reduction and massive gene decay due to loss of selection on gene functions, which is likely a consequence of having become an obligate mutualist of its tick host. Polymorphisms within the sampled CRt population implied the existence of several strain variants, and showed mutational patterns that might be a sign of selection on synonymous sites. Comparative genomics between the two tick symbionts CRt and CLEAA, showing the largest genome size difference between any two obligate endosymbionts from the same codiverging clade, indicates that CLEAA might experience an accelerated rate of evolution. We suggest that this might be a consequence of CLEAA loosing the genes *mutSL*, encoding the mismatch repair system. As both tick symbionts have kept many genes for B-vitamins synthesis, provisioning of these nutrients might be the function of *Coxiella*-like symbionts in ticks; however, further experiment might be needed to verify this observation.

## Supplementary Material

Supplementary figures S1–S6 and tables S1 and S2 are available at *Genome Biology and Evolution* online (http://www.gbe.oxfordjournals.org/).

## References

[evv108-B1] Akman L 2002 Genome sequence of the endocellular obligate symbiont of tsetse flies, *Wigglesworthia glossinidia*. Nat Genet. 32:402–407. 12219091 10.1038/ng986

[evv108-B2] Almeida AP 2012 *Coxiella* symbiont in the tick *Ornithodoros rostratus* (Acari: Argasidae). Ticks Tick Borne Dis. 3:203–206. 22480930 10.1016/j.ttbdis.2012.02.003

[evv108-B3] Babbitt GA Alawad MA Schulze KV Hudson AO 2014 Synonymous codon bias and functional constraint on GC3-related DNA backbone dynamics in the prokaryotic nucleoid. Nucleic Acids Res. 42:10915–10926. 25200075 10.1093/nar/gku811 PMC4176184

[evv108-B4] Bateman A 2004 The Pfam protein families database. Nucleic Acids Res. 32:D138–D141. 14681378 10.1093/nar/gkh121 PMC308855

[evv108-B5] Beare PA 2009 Comparative genomics reveal extensive transposon-mediated genomic plasticity and diversity among potential effector proteins within the genus *Coxiella*. Infect Immun. 77:642–656. 19047403 10.1128/IAI.01141-08 PMC2632050

[evv108-B6] Begley TP 1999 Thiamin biosynthesis in prokaryotes. Arch Microbiol. 171:293–300. 10382260 10.1007/s002030050713

[evv108-B7] Bennett GM McCutcheon JP MacDonald BR Romanovicz D Moran NA 2014 Differential genome evolution between companion symbionts in an insect-bacterial symbiosis. MBio 5:e01697-14. 10.1128/mBio.01697-14 PMC4196230 25271287

[evv108-B8] Benson G 1999 Tandem repeats finder: a program to analyze DNA sequences. Nucleic Acids Res. 27:573–580. 9862982 10.1093/nar/27.2.573 PMC148217

[evv108-B9] Bermingham A Derrick JP 2002 The folic acid biosynthesis pathway in bacteria: evaluation of potential for antibacterial drug discovery. BioEssays 24:637–648. 12111724 10.1002/bies.10114

[evv108-B10] Bolger AM Lohse M Usadel B 2014 Trimmomatic: a flexible trimmer for Illumina sequence data. Bioinformatics 30:2114–2120. 24695404 10.1093/bioinformatics/btu170 PMC4103590

[evv108-B11] Bourque G Pevzner PA 2002 Genome-scale evolution: reconstructing gene orders in the ancestral species. Genome Res. 12:26–36. 11779828 PMC155248

[evv108-B12] Braun V Hantke K 2011 Recent insights into iron import by bacteria. Curr Opin Chem Biol. 15:328–334. 21277822 10.1016/j.cbpa.2011.01.005

[evv108-B13] Bromham L 2009 Why do species vary in their rate of molecular evolution? Biol Lett.. 5:401–404. 19364710 10.1098/rsbl.2009.0136 PMC2679939

[evv108-B14] Buchner P. 1965 Endosymbiosis of animals with plant microorganisms. Interscience.

[evv108-B15] Burke GR Moran NA 2011 Massive genomic decay in *Serratia symbiotica*, a recently evolved symbiont of aphids. Genome Biol Evol. 3:195–208. 21266540 10.1093/gbe/evr002 PMC3056288

[evv108-B16] Chen DE Podell S Sauer J-D Swanson MS Saier MH 2008 The phagosomal nutrient transporter (Pht) family. Microbiology 154:42–53. 18174124 10.1099/mic.0.2007/010611-0

[evv108-B17] Childs JE Paddock CD 2003 The ascendancy of *Amblyomma americanum* as a vector of pathogens affecting humans in the United States. Annu Rev Entomol. 48:307–337. 12414740 10.1146/annurev.ento.48.091801.112728

[evv108-B18] Clay K 2008 Microbial communities and interactions in the lone star tick, *Amblyomma americanum*. Mol Ecol. 17:4371–4381. 19378409 10.1111/j.1365-294x.2008.03914.x

[evv108-B19] Cole ST 2001 Massive gene decay in the leprosy bacillus. Nature 409:1007–1011. 11234002 10.1038/35059006

[evv108-B20] Collins HL 2003 The role of iron in infections with intracellular bacteria. Immunol Lett. 85:193–195. 12527227 10.1016/s0165-2478(02)00229-8

[evv108-B21] Contreras H Chim N Credali A Goulding CW 2014 Heme uptake in bacterial pathogens. Curr Opin Chem Biol. 19:34–41. 24780277 10.1016/j.cbpa.2013.12.014 PMC4007353

[evv108-B22] Darling AC Mau B Blattner FR Perna NT 2004 Mauve: multiple alignment of conserved genomic sequence with rearrangements. Genome Res. 14:1394–1403. 15231754 10.1101/gr.2289704 PMC442156

[evv108-B23] de Crécy-Lagard V Marck C Grosjean H 2012 Decoding in *Candidatus Riesia pediculicola*, close to a minimal tRNA modification set? Trends Cell Mol Biol.. 7:11–34. 23308034 PMC3539174

[evv108-B24] Denison AM Massung RF Thompson HA 2007 Analysis of the O-antigen biosynthesis regions of phase II Isolates of *Coxiella burnetii*. FEMS Microbiol Lett. 267:102–107. 17156123 10.1111/j.1574-6968.2006.00544.x

[evv108-B25] Dunning Hotopp JC 2006 Comparative genomics of emerging human ehrlichiosis agents. PLoS Genet. 2:208–223. 10.1371/journal.pgen.0020021 PMC1366493 16482227

[evv108-B26] Duron O Jourdain E McCoy KD 2014 Diversity and global distribution of the *Coxiella* intracellular bacterium in seabird ticks. Ticks Tick Borne Dis. 5:557–563. 24915875 10.1016/j.ttbdis.2014.04.003

[evv108-B27] Ellegaard KM Klasson L Naslund K Bourtzis K Andersson SGE 2013 Comparative genomics of *Wolbachia* and the bacterial species concept. PLoS Genet. 9:e1003381. 23593012 10.1371/journal.pgen.1003381 PMC3616963

[evv108-B28] Fliermans CB 1996 Ecology of legionella: from data to knowledge with a little wisdom. Microb Ecol. 32:203–228. 8688009 10.1007/BF00185888

[evv108-B29] Fuchs TM Eisenreich W Heesemann J Goebel W 2012 Metabolic adaptation of human pathogenic and related nonpathogenic bacteria to extra- and intracellular habitats. FEMS Microbiol Rev. 36:435–462. 22092350 10.1111/j.1574-6976.2011.00301.x

[evv108-B30] Gophna U Thompson JR Boucher Y Doolittle WF 2006 Complex histories of genes encoding 3-hydroxy-3-methylglutaryl-coenzymeA reductase. Mol Biol Evol. 23:168–178. 16162862 10.1093/molbev/msj019

[evv108-B31] Gordon D Abajian C Green P 1998 Consed: a graphical tool for sequence finishing. Genome Res. 8:195–202. 9521923 10.1101/gr.8.3.195

[evv108-B32] Graca-Souza AV 2006 Adaptations against heme toxicity in blood-feeding arthropods. Insect Biochem Mol Biol. 36:322–335. 16551546 10.1016/j.ibmb.2006.01.009

[evv108-B33] Gruber S 2014 Multilayer chromosome organization through DNA bending, bridging and extrusion. Curr Opin Microbiol. 22:102–110. 25460803 10.1016/j.mib.2014.09.018

[evv108-B34] Guy L Kultima JR Andersson SG 2010 genoPlotR: comparative gene and genome visualization in R. Bioinformatics 26:2334–2335. 20624783 10.1093/bioinformatics/btq413 PMC2935412

[evv108-B35] Hackstadt TED 1990 The role of lipopolysaccharides in the virulence of *Coxiella burnetii*. Ann N Y Acad Sci. 590:27–32. 2378455 10.1111/j.1749-6632.1990.tb42203.x

[evv108-B36] Hager AJ 2006 Type IV pili-mediated secretion modulates *Francisella* virulence. Mol Microbiol. 62:227–237. 16987180 10.1111/j.1365-2958.2006.05365.x

[evv108-B37] Hansen AK Moran NA 2014 The impact of microbial symbionts on host plant utilization by herbivorous insects. Mol Ecol. 23:1473–1496. 23952067 10.1111/mec.12421

[evv108-B38] Hoover TA Culp DW Vodkin MH Williams JC Thompson HA 2002 Chromosomal DNA deletions explain phenotypic characteristics of two antigenic variants, Phase II and RSA 514 (Crazy), of the *Coxiella burnetii* nine mile strain. Infect Immun. 70:6726–6733. 12438347 10.1128/IAI.70.12.6726-6733.2002 PMC132984

[evv108-B39] Hosokawa T Koga R Kikuchi Y Meng X-Y Fukatsu T 2010 *Wolbachia* as a bacteriocyte-associated nutritional mutualist. Proc Natl Acad Sci U S A. 107:769–774. 20080750 10.1073/pnas.0911476107 PMC2818902

[evv108-B40] Hyatt D 2010 Prodigal: prokaryotic gene recognition and translation initiation site identification. BMC Bioinformatics 11:119. 20211023 10.1186/1471-2105-11-119 PMC2848648

[evv108-B41] Ioffe-Uspensky I Mumcuoglu KY Uspensky I Galun R 1997 *Rhipicephalus sanguineus* and *R. turanicus* (Acari: Ixodidae): closely related species with different biological characteristics. J Med Entomol. 34:74–81. 9086715 10.1093/jmedent/34.1.74

[evv108-B42] Kahramanoglou C 2012 Genomics of DNA cytosine methylation in *Escherichia coli* reveals its role in stationary phase transcription. Nat Commun. 3:886. 22673913 10.1038/ncomms1878

[evv108-B43] Katoh K Misawa K Kuma K Miyata T 2002 MAFFT: a novel method for rapid multiple sequence alignment based on fast Fourier transform. Nucleic Acids Res. 30:3059–3066. 12136088 10.1093/nar/gkf436 PMC135756

[evv108-B44] Kersh GJ 2010 Presence of *Coxiella burnetii* DNA in the environment of the United States, 2006 to 2008. Appl Environ Microbiol. 76:4469–4475. 20472727 10.1128/AEM.00042-10 PMC2897457

[evv108-B45] Kim H Lee H Shin D 2012 The FeoA protein is necessary for the FeoB transporter to import ferrous iron. Biochem Biophys Res Commun. 423:733–738. 22705302 10.1016/j.bbrc.2012.06.027

[evv108-B46] Kirkness EF 2010 Genome sequences of the human body louse and its primary endosymbiont provide insights into the permanent parasitic lifestyle. Proc Natl Acad Sci U S A. 107:12168–12173. 20566863 10.1073/pnas.1003379107 PMC2901460

[evv108-B47] Kisker C Kuper J Van Houten B 2013 Prokaryotic nucleotide excision repair. Cold Spring Harb Perspect Biol. 5:a012591. 23457260 10.1101/cshperspect.a012591 PMC3578354

[evv108-B48] Klyachko O Stein BD Grindle N Clay K Fuqua C 2007 Localization and visualization of a *Coxiella*-Type symbiont within the lone star tick, *Amblyomma americanum*. Appl Environ Microbiol. 73:6584–6594. 17720830 10.1128/AEM.00537-07 PMC2075054

[evv108-B49] Kurtz S 2004 Versatile and open software for comparing large genomes. Genome Biol. 5:R12. 14759262 10.1186/gb-2004-5-2-r12 PMC395750

[evv108-B50] Lagesen K 2007 RNAmmer: consistent and rapid annotation of ribosomal RNA genes. Nucleic Acids Res. 35:3100–3108. 17452365 10.1093/nar/gkm160 PMC1888812

[evv108-B51] Lalzar I Friedmann Y Gottlieb Y 2014 Tissue tropism and vertical transmission of *Coxiella* in *Rhipicephalus sanguineus* and *Rhipicephalus turanicus* ticks. Environ Microbiol. 16:3657–3668. 24650112 10.1111/1462-2920.12455

[evv108-B52] Lalzar I Harrus S Mumcuoglu KY Gottlieb Y 2012 Composition and seasonal variation of *Rhipicephalus turanicus* and *Rhipicephalus sanguineus* bacterial communities. Appl Environ Microbiol. 78:4110–4116. 22467507 10.1128/AEM.00323-12 PMC3370557

[evv108-B53] Lamelas A 2011 *Serratia symbiotica* from the aphid *Cinara cedri*: a missing link from facultative to obligate Insect endosymbiont. PLoS Genet. 7:e1002357. 22102823 10.1371/journal.pgen.1002357 PMC3213167

[evv108-B54] Lara FA Lins U Bechara GH Oliveira PL 2005 Tracing heme in a living cell: hemoglobin degradation and heme traffic in digest cells of the cattle tick *Boophilus microplus*. J Exp Biol. 208:3093–3101. 16081607 10.1242/jeb.01749

[evv108-B55] Lee H Popodi E Tang H Foster PL 2012 Rate and molecular spectrum of spontaneous mutations in the bacterium *Escherichia coli* as determined by whole-genome sequencing. Proc Natl Acad Sci U S A. 109:E2774–E2783. 22991466 10.1073/pnas.1210309109 PMC3478608

[evv108-B56] Leigh JA Dodsworth JA 2007 Nitrogen regulation in bacteria and archaea. Annu Rev Microbiol. 61:349–377. 17506680 10.1146/annurev.micro.61.080706.093409

[evv108-B57] Li H Durbin R 2009 Fast and accurate short read alignment with Burrows–Wheeler transform. Bioinformatics 25:1754–1760. 19451168 10.1093/bioinformatics/btp324 PMC2705234

[evv108-B58] Li L Stoeckert CJ Roos DS 2003 OrthoMCL: identification of ortholog groups for eukaryotic genomes. Genome Res. 13:2178–2189. 12952885 10.1101/gr.1224503 PMC403725

[evv108-B59] Liu L 2013 Coinfection of *Dermacentor silvarum* Olenev (Acari: Ixodidae) by *Coxiella*-like, *Arsenophonus*-like, and *Rickettsia*-like symbionts. Appl Environ Microbiol. 79:2450–2454. 23354701 10.1128/AEM.03575-12 PMC3623253

[evv108-B60] Lopez-Sanchez MJ 2009 Evolutionary convergence and nitrogen metabolism in Blattabacterium strain Bge, primary endosymbiont of the cockroach *Blattella germanica*. PLoS Genet. 5:e1000721. 19911043 10.1371/journal.pgen.1000721 PMC2768785

[evv108-B61] Lowe TM Eddy SR 1997 tRNAscan-SE: a program for improved detection of transfer RNA genes in genomic sequence. Nucleic Acids Res. 25:0955–0964. 10.1093/nar/25.5.955 PMC146525 9023104

[evv108-B62] Macdonald SJ Lin GG Russell CW Thomas GH Douglas AE 2012 The central role of the host cell in symbiotic nitrogen metabolism. Proc R Soc Lond B Biol Sci. 279:2965–2973. 10.1098/rspb.2012.0414 PMC3385485 22513857

[evv108-B63] Machado-Ferreira E 2011 *Coxiella* symbionts in the cayenne tick *Amblyomma cajennense*. Microb Ecol. 62:134–142. 21611689 10.1007/s00248-011-9868-x

[evv108-B64] Manzano-Marin A Latorre A 2014 Settling down: the genome of *Serratia symbiotica* from the aphid *Cinara tujafilina* zooms in on the process of accommodation to a cooperative intracellular life. Genome Biol Evol. 6:1683–1698. 24951564 10.1093/gbe/evu133 PMC4122931

[evv108-B65] Maurin M Raoult D 1999 Q fever. Clin Microbiol Rev. 12:518–553. 10515901 10.1128/cmr.12.4.518 PMC88923

[evv108-B66] Merhej V Royer-Carenzi M Pontarotti P Raoult D 2009 Massive comparative genomic analysis reveals convergent evolution of specialized bacteria. Biol Direct. 4:13. 19361336 10.1186/1745-6150-4-13 PMC2688493

[evv108-B67] Miller CN 2013 PanG, a new ketopantoate reductase involved in pantothenate synthesis. J Bacteriol. 195:965–976. 23243306 10.1128/JB.01740-12 PMC3571331

[evv108-B68] Moran NA 1996 Accelerated evolution and Muller’s rachet in endosymbiotic bacteria. Proc Natl Acad Sci U S A. 93:2873–2878. 8610134 10.1073/pnas.93.7.2873 PMC39726

[evv108-B69] Moran NA Bennett GM 2014 The tiniest tiny genomes. Annu Rev Microbiol. 68:195–215. 24995872 10.1146/annurev-micro-091213-112901

[evv108-B70] Moran NA McCutcheon JP Nakabachi A 2008 Genomics and evolution of heritable bacterial symbionts. Annu Rev Genet. 42:165–190. 18983256 10.1146/annurev.genet.41.110306.130119

[evv108-B71] Moriya Y Itoh M Okuda S Yoshizawa AC Kanehisa M 2007 KAAS: an automatic genome annotation and pathway reconstruction server. Nucleic Acids Res. 35:W182–W185. 17526522 10.1093/nar/gkm321 PMC1933193

[evv108-B72] Mott ML Berger JM 2007 DNA replication initiation: mechanisms and regulation in bacteria. Nat Rev Microbiol. 5:343–354. 17435790 10.1038/nrmicro1640

[evv108-B73] Mudrow E 1932 Über die intrazellulären symbionten der zecken. Z Parasitenkd. 5:138–183.

[evv108-B74] Mumcuoglu KY Burgan I Ioffe-Uspensky I Manor O 1993 *Rhipicephalus sanguineus*: observations on the parasitic stage on dogs in the Negev Desert of Israel. Exp Appl Acarol. 17:793–798. 7628225 10.1007/BF00225852

[evv108-B75] Nikoh N 2014 Evolutionary origin of insect–*Wolbachia* nutritional mutualism. Proc Natl Acad Sci U S A. 111:10257–10262. 24982177 10.1073/pnas.1409284111 PMC4104916

[evv108-B76] Nilsson AI 2005 Bacterial genome size reduction by experimental evolution. Proc Natl Acad Sci U S A. 102:12112–12116. 16099836 10.1073/pnas.0503654102 PMC1189319

[evv108-B77] Noda H Munderloh UG Kurtti TJ 1997 Endosymbionts of ticks and their relationship to *Wolbachia *spp. and tick-borne pathogens of humans and animals. Appl Environ Microbiol. 63:3926–3932. 9327557 10.1128/aem.63.10.3926-3932.1997 PMC168704

[evv108-B78] Noguchi S Nishimura Y Hirota Y Nishimura S 1982 Isolation and characterization of an *Escherichia coli* mutant lacking tRNA-guanine transglycosylase. Function and biosynthesis of queuosine in tRNA. J Biol Chem. 257:6544–6550. 6804468

[evv108-B79] Omsland A Heinzen RA 2011 Life on the outside: the rescue of *Coxiella burnetii* from its host cell. Annu Rev Microbiol. 65:111–128. 21639786 10.1146/annurev-micro-090110-102927

[evv108-B80] Padan E Maisler N Taglicht D Karpel R Schuldiner S 1989 Deletion of ant in *Escherichia coli* reveals its function in adaptation to high salinity and an alternative Na+/H+ antiporter system(s). J Biol Chem. 264:20297–20302. 2555351

[evv108-B81] Pati A 2010 GenePRIMP: a gene prediction improvement pipeline for prokaryotic genomes. Nat Methods. 7:455–457. 20436475 10.1038/nmeth.1457

[evv108-B82] Pegram RG Clifford CM Walker J Keirans JE 1987 Clarification of the *Rhipicephalus sanguineus* group (Acari, Ixodoidea, Ixodidae). I. *R. sulcatus* Neumann, 1908 and *R. turanicus* Pomerantsev, 1936. Syst Parasitol. 10:3–26.

[evv108-B83] Petit MA Dimpfl J Radman M Echols H 1991 Control of large chromosomal duplications in *Escherichia coli* by the mismatch repair system. Genetics 129:327–332. 1743481 10.1093/genetics/129.2.327 PMC1204626

[evv108-B84] Ramsey JS 2010 Genomic evidence for complementary purine metabolism in the pea aphid, *Acyrthosiphon pisum*, and its symbiotic bacterium *Buchnera aphidicola*. Insect Mol Biol. 19:241–248. 20482654 10.1111/j.1365-2583.2009.00945.x

[evv108-B85] Ran W Higgs PG 2010 The influence of anticodon–codon interactions and modified bases on codon usage bias in bacteria. Mol Biol Evol. 27:2129–2140. 20403966 10.1093/molbev/msq102

[evv108-B86] Reumers J 2005 SNPeffect: a database mapping molecular phenotypic effects of human non-synonymous coding SNPs. Nucleic Acids Res. 33:D527–D532. 15608254 10.1093/nar/gki086 PMC540040

[evv108-B87] Roden JA Wells DH Chomel BB Kasten RW Koehler JE 2012 Hemin binding protein C is found in outer membrane vesicles and protects *Bartonella henselae* against toxic concentrations of hemin. Infect Immun. 80:929–942. 22232189 10.1128/IAI.05769-11 PMC3294634

[evv108-B88] Roux V Bergoin M Lamaze N Raoult D 1997 Reassessment of the taxonomic position of *Rickettsiella grylli*. Int J Syst Bacteriol. 47:1255–1257. 9336939 10.1099/00207713-47-4-1255

[evv108-B89] Rutherford K 2000 Artemis: sequence visualization and annotation. Bioinformatics 16:944–945. 11120685 10.1093/bioinformatics/16.10.944

[evv108-B90] Sabat A 2003 New method for typing *Staphylococcus aureus* strains: multiple-locus variable-number tandem repeat analysis of polymorphism and genetic relationships of clinical isolates. J Clin Microbiol. 41:1801–1804. 12682193 10.1128/JCM.41.4.1801-1804.2003 PMC153872

[evv108-B91] Sabree ZL Kambhampati S Moran NA 2009 Nitrogen recycling and nutritional provisioning by Blattabacterium, the cockroach endosymbiont. Proc Natl Acad Sci U S A. 106:19521–19526. 19880743 10.1073/pnas.0907504106 PMC2780778

[evv108-B92] Schaible UE Kaufmann SHE 2004 Iron and microbial infection. Nat Rev Microbiol. 2:946–953. 15550940 10.1038/nrmicro1046

[evv108-B93] Schofield MJ Hsieh P 2003 DNA mismatch repair: molecular mechanisms and biological function. Annu Rev Microbiol. 57:579–608. 14527292 10.1146/annurev.micro.57.030502.090847

[evv108-B94] Segal G Feldman M Zusman T 2005 The Icm/Dot type-IV secretion systems of *Legionella pneumophila* and *Coxiella burnetii*. FEMS Microbiol Rev. 29:65–81. 15652976 10.1016/j.femsre.2004.07.001

[evv108-B95] Seshadri R 2003 Complete genome sequence of the Q-fever pathogen *Coxiella burnetii*. Proc Natl Acad Sci U S A. 100:5455–5460. 12704232 10.1073/pnas.0931379100 PMC154366

[evv108-B96] Seshadri R Samuel J 2005 Genome analysis of *Coxiella burnetii* species: insights into pathogenesis and evolution and implications for biodefense. Ann N Y Acad Sci. 1063:442–450. 16481558 10.1196/annals.1355.063

[evv108-B97] Sharp PM Li WH 1987 The rate of synonymous substitution in enterobacterial genes is inversely related to codon usage bias. Mol Biol Evol. 4:222–230. 3328816 10.1093/oxfordjournals.molbev.a040443

[evv108-B98] Simpson JT Durbin R 2012 Efficient de novo assembly of large genomes using compressed data structures. Genome Res. 22:549–556. 22156294 10.1101/gr.126953.111 PMC3290790

[evv108-B99] Simpson JT 2009 ABySS: a parallel assembler for short read sequence data. Genome Res. 19:1117–1123. 19251739 10.1101/gr.089532.108 PMC2694472

[evv108-B100] Smith TA Driscoll T Gillespie JJ Raghavan R 2015 A *Coxiella*-like endosymbiont is a potential vitamin source for the Lone Star tick. Genome Biol Evol. 7:831–838 25618142 10.1093/gbe/evv016 PMC4994718

[evv108-B101] Snyder AK Deberry JW Runyen-Janecky L Rio RVM 2010 Nutrient provisioning facilitates homeostasis between tsetse fly (Diptera: Glossinidae) symbionts. Proc R Soc Lond B Biol Sci. 277:2389–2397. 10.1098/rspb.2010.0364 PMC2894912 20356887

[evv108-B102] Sonenshine DE Roe RM 2014 Biology of ticks. New York: Oxford University Press.

[evv108-B103] Sprong H 2012 Prevalence of *Coxiella burnetii* in ticks after a large outbreak of Q fever. Zoonoses Public Health. 59:69–75. 21824373 10.1111/j.1863-2378.2011.01421.x

[evv108-B104] Stamatakis A 2006 RAxML-VI-HPC: maximum likelihood-based phylogenetic analyses with thousands of taxa and mixed models. Bioinformatics 22:2688–2690. 16928733 10.1093/bioinformatics/btl446

[evv108-B105] Stead C Omsland A Beare P Sandoz K Heinzen R 2013 Sec-mediated secretion by *Coxiella burnetii*. BMC Microbiology 13:222. 24093460 10.1186/1471-2180-13-222 PMC3882888

[evv108-B106] Toft C Andersson SGE 2010 Evolutionary microbial genomics: insights into bacterial host adaptation. Nat Rev Genet. 11:465–475. 20517341 10.1038/nrg2798

[evv108-B107] Toh H 2006 Massive genome erosion and functional adaptations provide insights into the symbiotic lifestyle of *Sodalis glossinidius* in the tsetse host. Genome Res. 16:149–156. 16365377 10.1101/gr.4106106 PMC1361709

[evv108-B108] Troughton DR Levin ML 2007 Life cycles of seven ixodid tick species (Acari : Ixodidae) under standardized laboratory conditions. J Med Entomol. 44:732–740. 17915502 10.1603/0022-2585(2007)44[732:lcosit]2.0.co;2

[evv108-B109] van Ham RCHJ 2003 Reductive genome evolution in *Buchnera aphidicola*. Proc Natl Acad Sci U S A. 100:581–586. 12522265 10.1073/pnas.0235981100 PMC141039

[evv108-B110] Van Leuven JT McCutcheon JP 2012 An AT mutational bias in the tiny GC-rich endosymbiont genome of *Hodgkinia*. Genome Biol Evol. 4:24–27. 22113795 10.1093/gbe/evr125 PMC3267392

[evv108-B111] van Schaik EJ Chen C Mertens K Weber MM Samuel JE 2013 Molecular pathogenesis of the obligate intracellular bacterium *Coxiella burnetii*. Nat Rev Microbiol. 11:561–573. 23797173 10.1038/nrmicro3049 PMC4134018

[evv108-B112] Vavre F Braig HR 2012 Primary and secondary symbionts, so similar, yet so different. In: Zchori-Fein E Bourtzis K, editors. Manipulative tenants: bacteria associated with arthropods. Boca Raton (FL): CRC Press p. xvii–xxxvii.

[evv108-B113] Vodkin MH Williams JC 1986 Overlapping deletion in two spontaneous phase variants of *Coxiella burnetii*. J Gen Microbiol. 132:2587–2594. 3794655 10.1099/00221287-132-9-2587

[evv108-B114] Wald N Alroy M Botzman M Margalit H 2012 Codon usage bias in prokaryotic pyrimidine-ending codons is associated with the degeneracy of the encoded amino acids. Nucleic Acids Res. 40:7074–7083. 22581775 10.1093/nar/gks348 PMC3424539

[evv108-B115] Weller C Wu M 2015 A generation-time effect on the rate of molecular evolution in bacteria. Evolution 69:643–652. 25564727 10.1111/evo.12597

[evv108-B116] Wilkinson DA 2014 Massive infection of seabird ticks with bacterial species related to *Coxiella burnetii*. Appl Environ Microbiol. 80:3327–3333. 24657860 10.1128/AEM.00477-14 PMC4018846

[evv108-B117] Williams LE Wernegreen JJ 2012 Purifying selection, sequence composition, and context-specific indel mutations shape intraspecific variation in a bacterial endosymbiont. Genome Biol Evol. 4:44–51. 22117087 10.1093/gbe/evr128 PMC3268670

[evv108-B118] Woolfit M Bromham L 2003 Increased rates of sequence evolution in endosymbiotic bacteria and fungi with small effective population sizes. Mol Biol Evol. 20:1545–1555. 12832648 10.1093/molbev/msg167

[evv108-B119] Yang Y Zhao GS Man TK Winkler ME 1998 Involvement of the gapA- and epd (gapB)-encoded dehydrogenases in pyridoxal 5′-phosphate coenzyme biosynthesis in *Escherichia coli* K-12. J Bacteriol. 180:4294–4299. 9696782 10.1128/jb.180.16.4294-4299.1998 PMC107430

[evv108-B120] Zerbino DR Birney E 2008 Velvet: algorithms for de novo short read assembly using de Bruijn graphs. Genome Res. 18:821–829. 18349386 10.1101/gr.074492.107 PMC2336801

[evv108-B121] Zhong J Jasinskas A Barbour AG 2007 Antibiotic treatment of the tick vector *Amblyomma americanum* reduced reproductive fitness. PLoS One. 2:e405 17476327 10.1371/journal.pone.0000405 PMC1852332

